# Biphalangeal/triphalangeal fifth toe and impact in the pathology of the fifth ray

**DOI:** 10.1186/1471-2474-15-295

**Published:** 2014-09-05

**Authors:** Jose Gallart, David González, Jose Valero, Javier Deus, Pedro Serrano, Manuel Lahoz

**Affiliations:** Department of Human Anatomy and Histology. School of Medicine, University of Zaragoza, Calle Domingo Miral s/n, Zaragoza, 50009 Spain; Department of Surgery, Obstetrics and Gynecology, School of Medicine, University of Zaragoza, Zaragoza, Spain; Department of Physiology, School of Medicine, University of Zaragoza, Zaragoza, Spain

**Keywords:** Fifth toe, Foot, Biphalangeal, Triphalangeal, Pathology, Hammer toe, Tailor’s bunion

## Abstract

**Background:**

Having reviewed the studies on the biphalangeal fifth toe, we have observed a great disparity of data depending on the research center. We have investigated the frequency of biphalangeal toes and also its handedness. We have also analyzed the relationship of pathological deviations of fifth toe with this feature and with the fifth metatarsal.

**Methods:**

We performed a descriptive prospective study, which analyzed 2494 feet (1247 people) with bilateral dorsoplantar radiographs. We studied the number of phalanges of the fifth toe, the deviations in the sagittal and transverse plane, and the state of the fifth metatarsal phalangeal joint.

**Results:**

After analyzing the data we found the presence of biphalangeal fifth toe in 46.3% of the feet, presenting this feature bilaterally in 97.4% of them. A statistically significant higher percentage of pathological toes was found in people with triphalangeal fifth toe (pathological in 29.91%) than in the biphalangeal toes (pathological in 15.60%). We found that these differences are accentuated in the alterations of the fifth toe in the sagittal plane.

**Conclusions:**

It is almost 4 times more likely to suffer a fifth hammer toe if the fifth toe is triphalangeal (OR = 3.98 to p =0.000). Alterations in the coronal plane of the fifth toe are associated with tailor’s bunion (p =0.000). We did not find any significant differences regarding the need for surgery of the fifth toe of the biphalangeal (39.1%) versus triphalangeal toes (60.9%). Clinical relevance: There may be an association between pathologic deviations and bigger mobility of the triphalangeal fifth toes. However, biphalangeal fifth toes show bigger rigidity leading to smaller accommodation inside the shoe, which may lead to less painful feet and decreased proportion of surgery.

**Electronic supplementary material:**

The online version of this article (doi:10.1186/1471-2474-15-295) contains supplementary material, which is available to authorized users.

## Background

**Figure 1:**
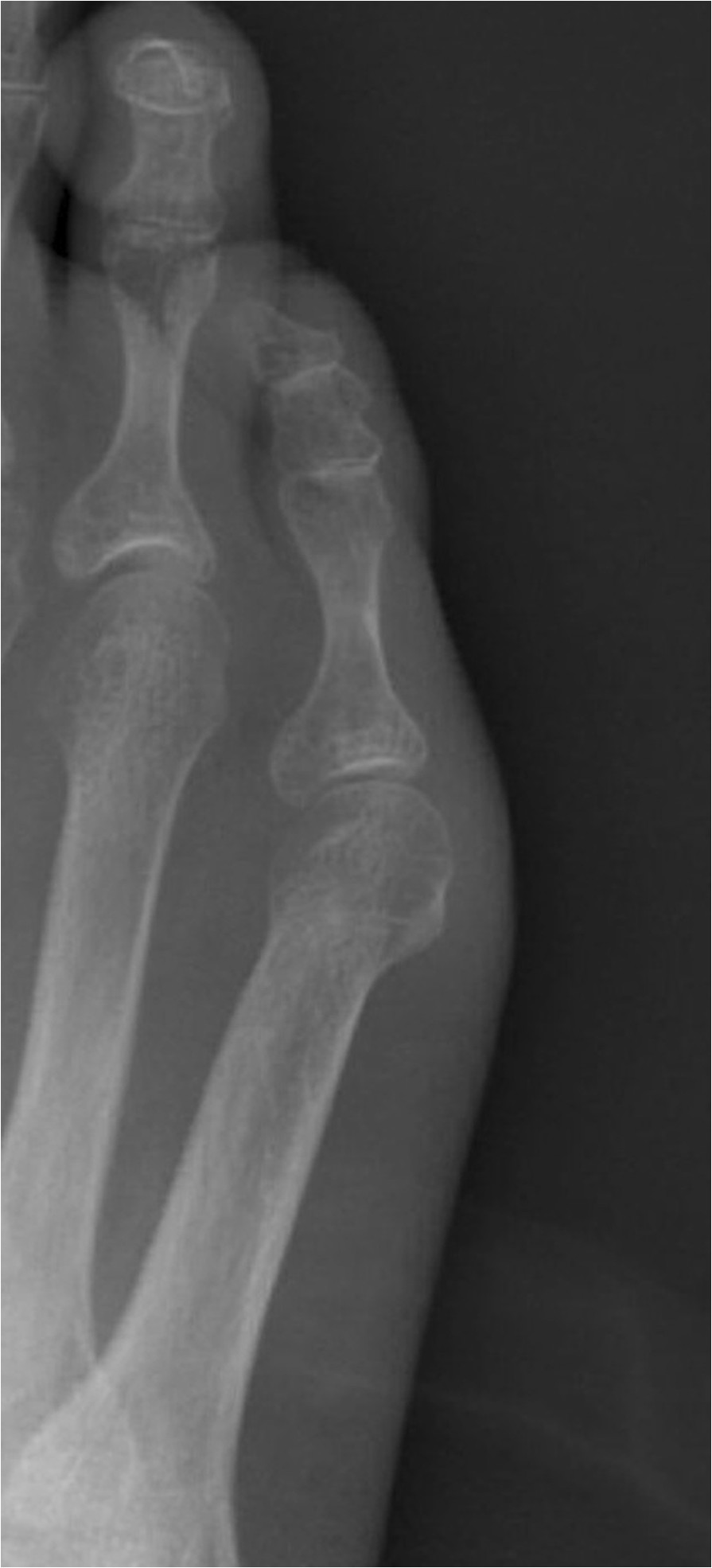
**Triphalangeal fifth toe.**

**Figure 2:**
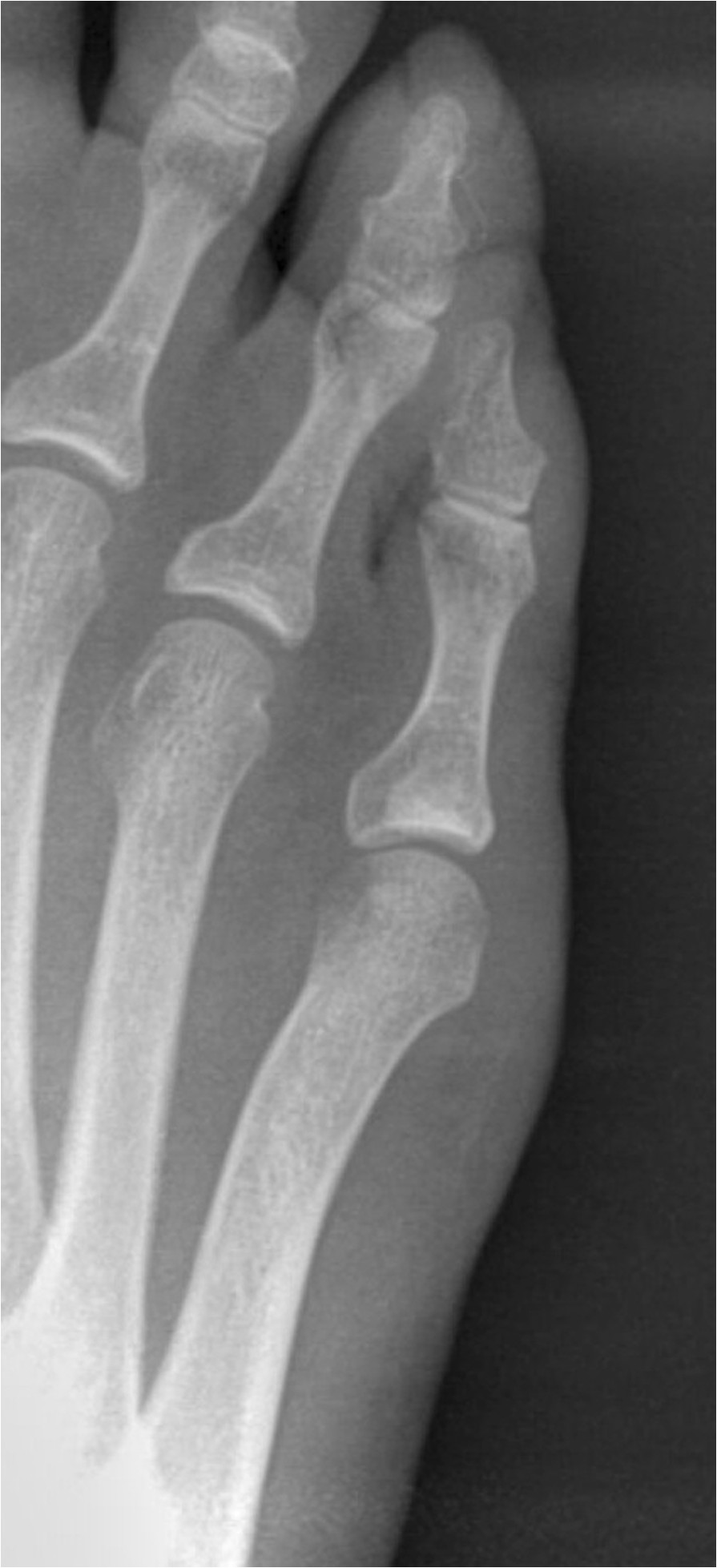
**Biphalangeal fifth toe.**

The biphalangeal fifth toe is described since ancient times. In his anatomical plates, Leonardo da Vinci represented the fifth toe with two phalanges [1]. However, Andrea Vesalius [2] represented the fifth toe with three phalanges clearly differentiated. Moreover, historically alterations in the number of phalanges in other toes, in isolation or multiple, are presented less frequently than in the fifth toe (Figures 1, 2).

Although the subject under study is not common in the international scientific literature, relatively recent references can be obtained, dealing with triphalangeal or biphalangeal fifth toe. Some authors have considered the issue is related to ethnicity, since the biphalangeal fifth toe is less common among African-Americans [3] while it is extremely common among the Japanese and Asian people, being found in more than 70% of the population [4, 5]. This anatomical peculiarity should be assessed in a radiological examination of the fifth toe when it requires surgery or is fractured [6].

The forms of anatomical presentation of this are manifold: unilateral or bilateral, in adults and children.

The aim of our research was:

1. To determine the frequency of triphalangeal and biphalangeal fifth toes.
2. Study of laterality (unilateral or bilateral) of this morphological feature.
3. Check if having two or three phalanges influences the pathology of the fifth toe or the fifth metatarsal.
  3.1 : To study the pathological deviation of the fifth toe in the sagittal and transverse planes, according to the number of phalanges.
  3.2 : To study the pathological deviation of the fifth foot ray, according to the number of phalanges.
4. Evaluate the presence of sesamoids under the fifth metatarsal head.

## Methods

**Figure 3:**
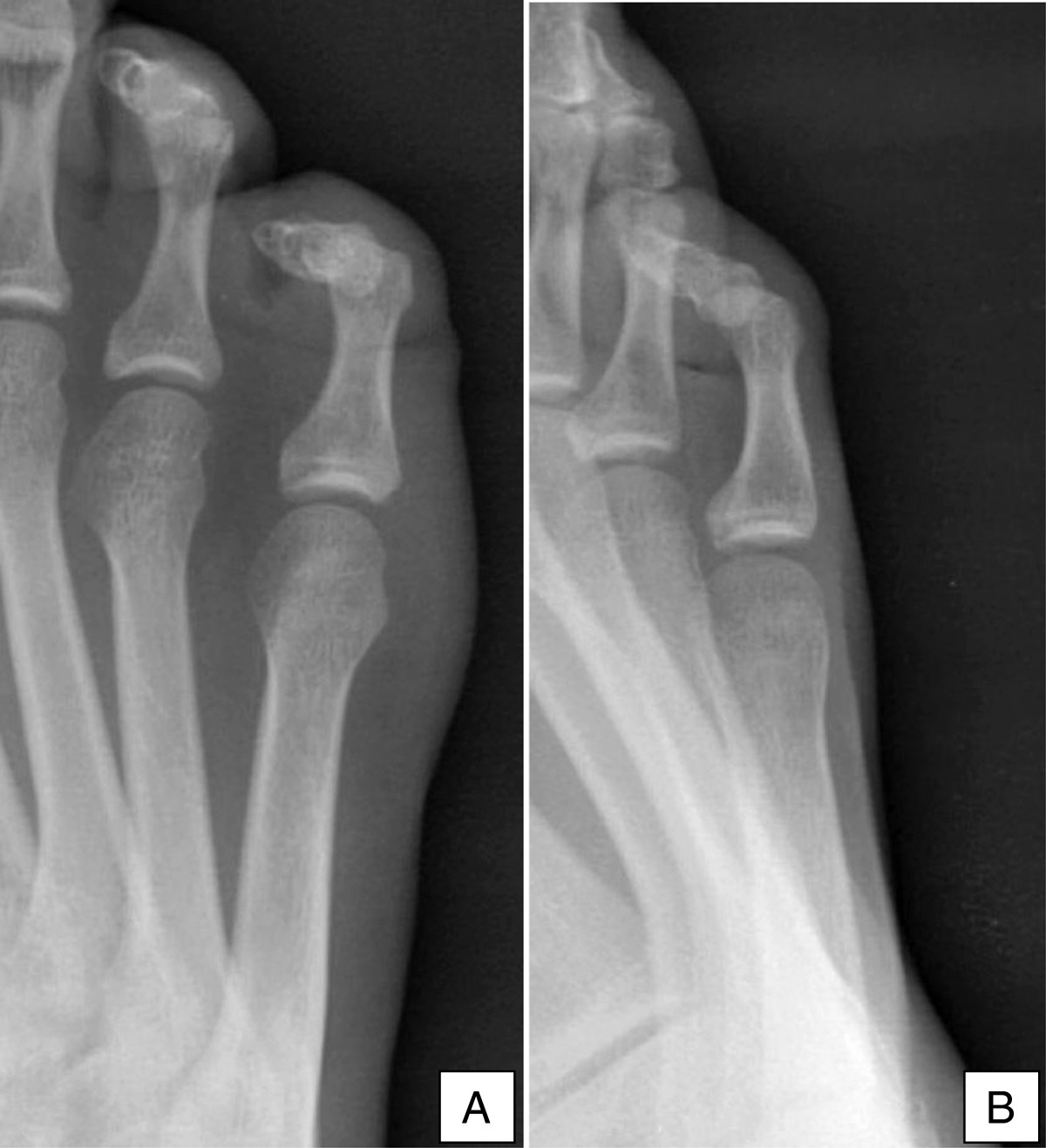
**Radiograph showing number of phalanges. (A)** Dorsoplantar radiograph showing inconclusive number of phalanges **(B)** Internal oblique radiograph of the same foot conclusively showing that the fifth toe is bifalangeal.

Before patients were included in the study, we obtained oral and written informed consent and explained to them the study protocol. We performed a prospective descriptive study, following the guidelines of the Declaration STROBE for observational studies, where the sample consisted of 2494 feet from 1247 individuals (all Caucasian), of which 173 were men (13.9%) and 1074 women (85.1%). We included in our study all the patients who consulted due to pain in the feet and were performed bilateral radiologic study in Dr. José Valero Clinic (Zaragoza, Spain) during three consecutive years (from April 1st 2007 to May 1st 2010). Ethics approval was not required because a purely observational, non-interactive study was carried out without interference in standard usual care and in accordance with normal practice and approvals.We used dorsoplantar bilateral projections previously performed for any clinical purposes, since the projection is suitable for morphological and pathological evaluation of the fifth toe and fifth ray, allowing assessing the bilateralism of the studied variables. In addition, we performed internal or external oblique projections when the dorsoplantar projection did not permit an optimal visualization of the fifth toe phalanges. Figure 3A shows a dorsoplantar radiograph with an inconclusive number of phalanges in a hammer fifth toe. On the other hand, Figure 3B demonstrates by means of an internal oblique projection that the fifth toe is biphalangeal.

The radiographic projections were made using a uniform pattern: the patient’s feet were together on the same chassis, the X-ray tube was located one meter away and tilted 15 degrees from the vertical and centered between the navicular bones of both feet. The radiographic parameters used were 60 kV power, 40 mA current and 0.1 seconds. All the patients were performed a clinical exploration to evaluate the fifth toe and the fifth ray.

We assessed the following variables:

1. Number of phalanges of the fifth toe.
2. Laterality.
3. Pathological deviations of the fifth toe in the sagittal and transverse planes. Adduct fifth toe was considered when the angle of the fifth metatarsophalangeal joint was greater than 10 degrees [7].

We considered pathology in the sagittal plane (fifth hammer toe/claws toe) if during the evaluation of the proximal interphalangeal joint, it was in plantarflexion, regardless the state of the distal interphalangeal joint.

We did not consider the pathology in the frontal plane, since the great majority of the pathology of the fifth toe is accompanied by varum rotation.

4. Presence or absence of pathology in the fifth metatarsal. We considered the existence of tailor’s bunion when the angle between the fourth and fifth metatarsal was greater than 10° degrees and/or there was deviation of the fifth metatarsal distal third greater than 8° degrees [8]. Presence or absence of sesamoids under the head of the fifth metatarsal.

Data regarding sex and previous surgeries performed in the fifth toe and fifth metatarsal were collected from the medical records of Dr. José Valero Clinic.

The data were analyzed with SPSS 17.0 statistic package. We considered the total sample of the study as the number of feet. We performed a frequency analysis and we used chi-square test to assess qualitative variables differences, assuming statistical significance when p-value was <0.05. The study was performed in agreement with the directives given in the Helsinki Declaration as revised in 1975 [9].

## Results

Of the 2494 feet studied, corresponding to 1247 individuals, we observed the presence of 3 phalanges in the fifth toe in 1339 feet (53.7%) and two phalanges in the fifth toe in 1155 (46.3%), showing this feature bilaterally in 2430 feet (97.4%) of the 46.3%. The presence of two phalanges in the fourth toe occurred in 64 cases (2.56%).

**Figure 4:**
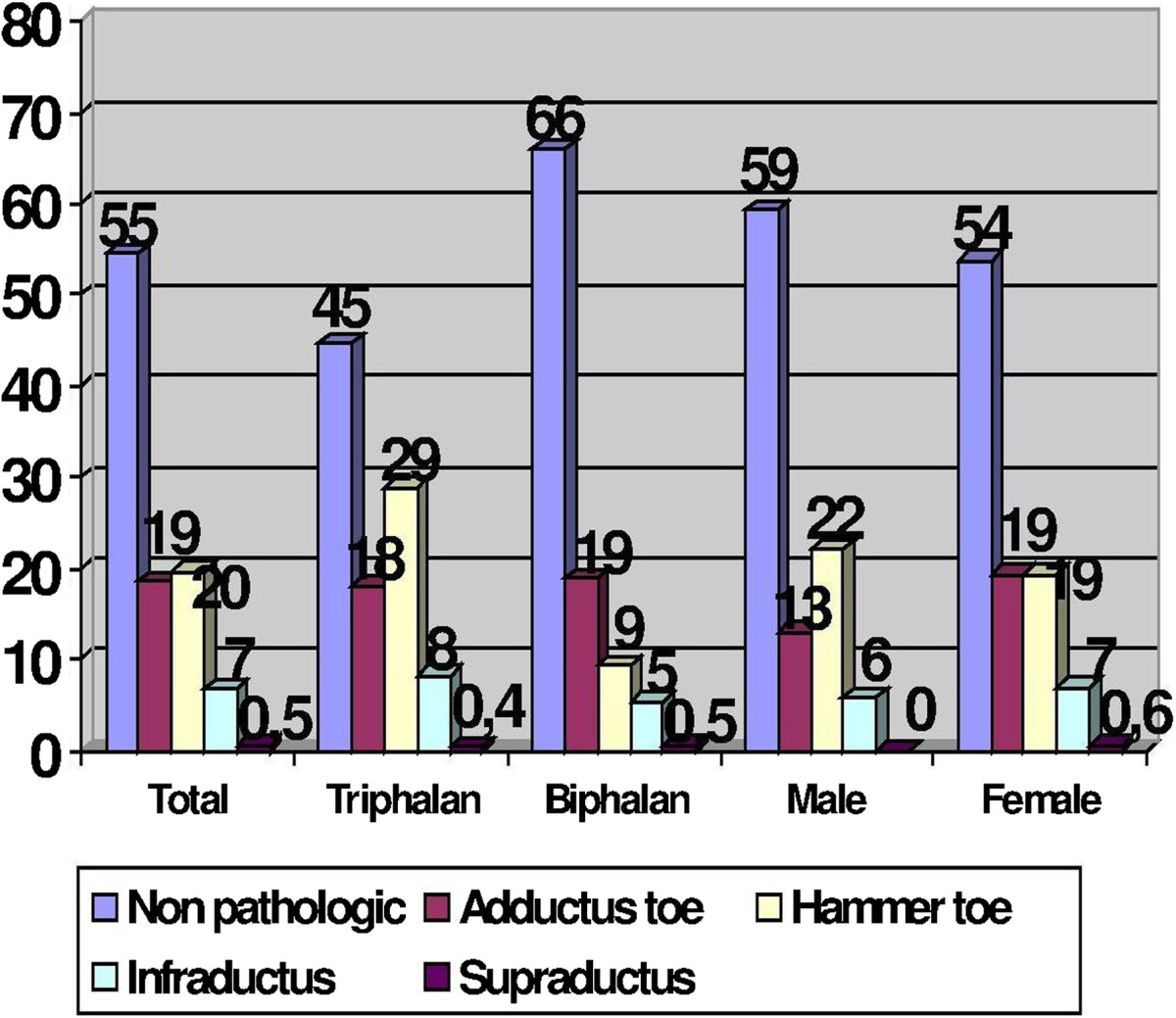
**Bar diagram showing the percentage of the various types of digital pathology according to the presence of triphalangeal or biphalangeal toes and also according to sex.**

Among the 346 feet of men, in 208 feet (60.12%) the fifth toe was triphalangeal and in 138 feet (39.88%) was biphalangeal. On the other hand, among the 2148 feet of women, in 1131 feet (52.65%) the fifth toe was triphalangeal, and in 1017 feet (47.35%) the fifth toe was biphalangeal. The differences in the proportion of biphalangeal and triphalangeal toes regarding the sex were statistically significant (p = 0.01), showing greater proportion of biphalangeal toes among females compared to males.With regard to the pathology of the fifth toe, it was recorded that 1359 (54.5%) toes did not present with any pathologic toes. We found 492 feet (19.7%) presenting with fifth hammer toe, 461 toes (18.5%) had toe deviation in adduction, 170 (6.8%) in infraductus toes and 12 (0.5%) in supraductus toes (Figure 4).Comparing the frequencies of the various pathologies of the fifth toe in relation to the presence of two or three phalanges, by chi-square test, we observed a significant higher percentage of pathological toe in people with fifth triphalangeal toe (pathological in 29.91%) than in people with biphalangeal toes (15.60%) (Figure 4).We discovered differences regarding the type of pathology in the toes (Figure 4): an increase in the proportion of hammer toes, and 4 times greater risk for hammer toe (OR = 3.98; 95% CI (3.15, 5.05); p = 0.000) among individuals with triphalangeal fifth toe as compared with biphalangeal fifth toe.

However, we did not find significant differences in the pathology of the fifth toe on the transverse plane (adductus toe) between triphalangeal and biphalangeal toes. Noting that these toes are influenced by the situation of the fifth metatarsophalangeal joint, and given that, when analyzing the toes that had pathology according to the state of the fifth metatarsophalangeal joint, we appreciated that there were significant differences (p = 0.000) and the majority of fifth adducts toes occurred in patients with tailor’s bunion. Of the 461 adduct or adduct varus toes, 371 (80.5%) presented in patients with tailor’s bunion.

Among the 2494 feet in our study, 69 feet were operated on the fifth toe (2.8% of the studied feet and 5.6% of the feet presenting with pathological deviations). Among the operated feet, 42 feet (60.9%) presented triphalangeal fifth toe and 27 (39.1%) presented biphalangeal fifth toe. In addition, 59 (85.5%) were from women and 10 (14.5%) from men.

With regards to the pathology of the fifth toe in the patients who had been operated on, 21 (30.4%) toes presented adductus varus, 44 (63.8%) presented hammer toe and 4 (5.8%) presented infraductus.

**Figure 5:**
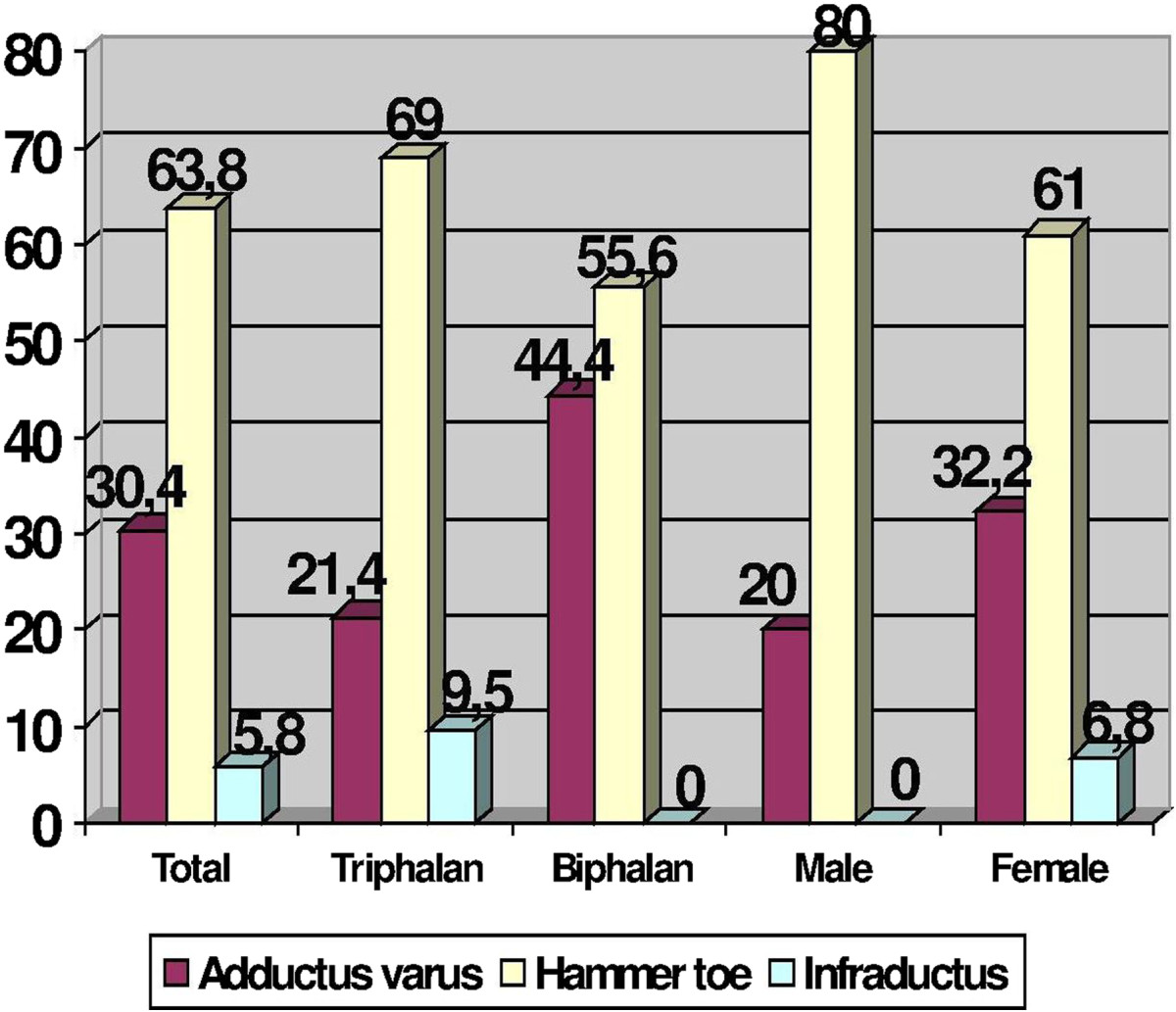
**Bar diagram showing the percentage of cases operated on, according to the presence of triphalangeal or biphalangeal toes and also according to sex.**

**Figure 6:**
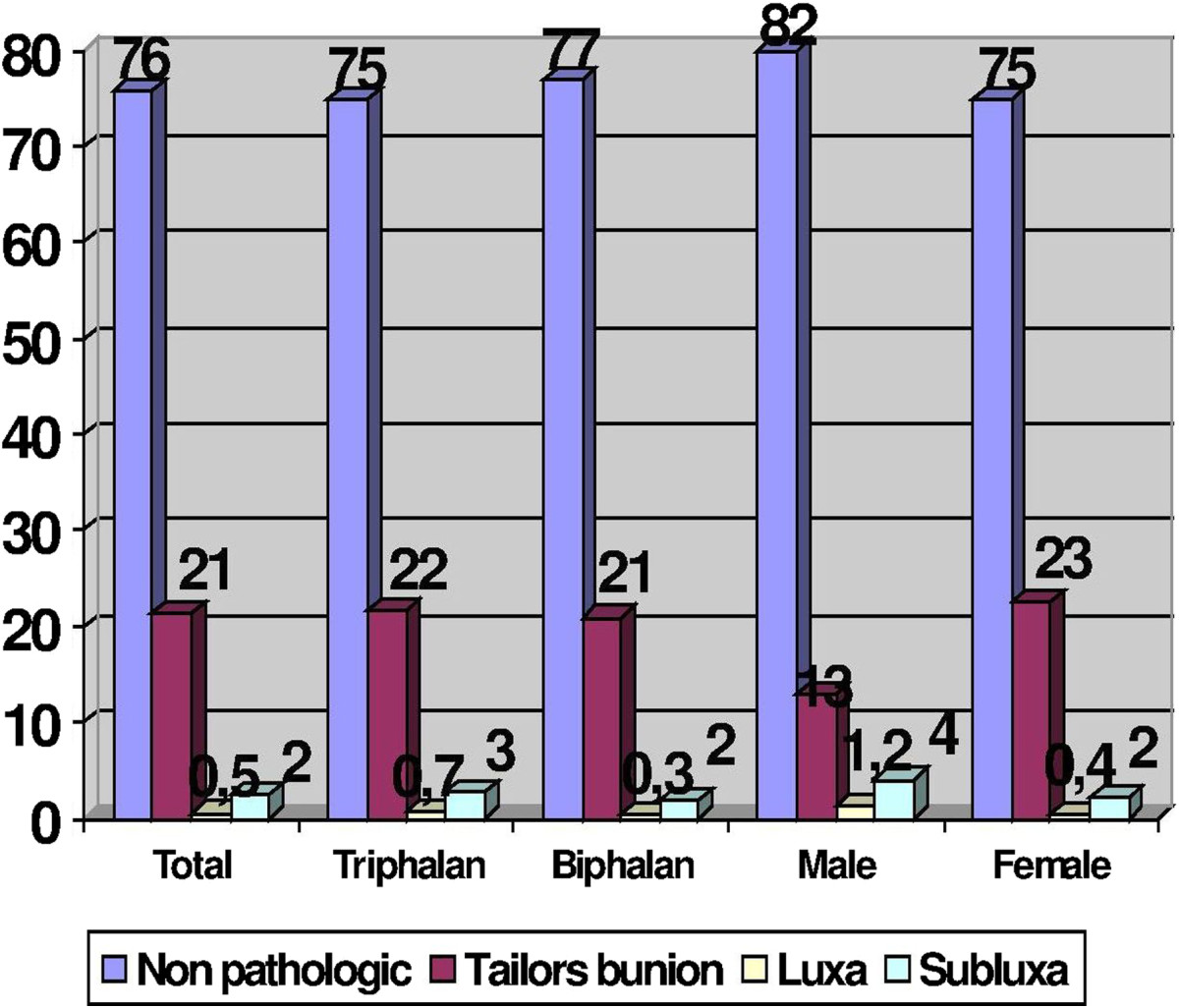
**Bar diagram showing the percentage of the various types of metatarsal pathology according to the presence of triphalangeal or biphalangeal toes and also according to sex.**

Among the 44 patients who were operated on due to hammer toe, 29 (65.9%) showed triphalangeal fifth toe and 15 (34.1%) showed biphalangeal fifth toe. On the other hand, among the 21 patients who were operated on due to adductus varus toe, 9 (42.9%) showed triphalangeal fifth toe and 12 (57.1%) showed biphalangeal fifth toe. Regarding sex, 14.5% of operated finger were from males and 85.5% were from women.Figure 5 shows, in the population of operated toes, the percentage of pathology according to tri or biphalangeal toes and also according to sex.With regard to the pathology of the fifth metatarsal, we found 1891 feet (75.8%) with no pathology, 530 feet (21.3%) with a tailor’s bunion, 13 feet (0.5%) with complete dislocation and 60 feet (2.4%) with subluxation in the sagittal plane fifth metatarsal phalangeal joint (Figure 6).

We found the presence of one or two accessory sesamoids under the fifth metatarsal head in 242 feet (9.7%). No significant differences were found between the presence of accessory sesamoids and pathology of the fifth metatarsal phalangeal joint.

## Discussion

The percentage of biphalangeal fifth toes observed in our study (46.3%) indicates that this is a common finding [10]. In our population, the rate is slightly higher than rates reported in Caucasians by others, such as Trolle and Aagesen [11]. They studied 370 feet of Danish and found 36% of biphalangeal fifth toes. In 1971 Sandström and Hedman [12] analyzed 496 Swedish people finding that 35% of them had biphalangia of the fifth toe. Le Minor in 1995 [3] studied French adults observing a prevalence of 41.02%. Compared to other studies conducted over years, the percentage of our study appears higher, and is very similar to the study conducted in 2012 by Moulton et al. [13] in which they found that 45% of the 655 feet analyzed had biphalangeal toes.

Some studies found a bigger proportion of biphalangeal fifth toe (60-65%) in a sample of fifth toes that were operated on and assumed that the rigidities caused by biphalangeal toes may lead to a significant increase in the pathology of the fifth toe and the fifth metatarsal [14, 15]. However, in our total population we have found an almost double percentage of pathological toes in people with fifth triphalangeal toe than in the biphalangeal toes 55.4% vs. 34%. We think that the higher motility of the fifth toe may lead to more pathology. Looking at the differences between toe pathologies these included a further increase in the proportion of hammer toes among individuals with triphalangeal fifth toes with respect to the biphalangeal fifth toes. In our population if the toe was triphalangeal, the risk of having a hammer toe was almost 4 times greater.

The explanation that may bring to an agreement both studies might be the following: In the first studies, they analyzed only patients who were operated on due to pathology in the fifth toe, but in our study we analyzed all the patients who consulted due to pain in the feet, either that were operated on or not. We only operated 2.3% of the sample and the proportion of interventions in triphalangeal fifth toes was 60.9%.

We argue that the triphalangeal fifth toes show bigger mobility and are more prone to pathologic deviations. However, biphalangeal fifth toes show bigger rigidity leading to smaller accommodation inside the shoe, which may lead to less painful feet and decreased proportion of surgery.

Although our sample there were significant differences in triphalangeal or biphalangeal fifth toes by gender, we believe that this fact could be affected by such an unequal sex proportion of our sample, 13.9% of men versus 85.1% of women, and also because the X-rays were carried out by suspected diagnosis of osteoarticular pathology in the foot. We believe that the frequency of pathology of the toes and the fifth metatarsal ray of this study may be conditioned by this fact.

Regarding the presence of one or more sesamoids under the head of the fifth metatarsal (9.7% of cases in our population), we have obtained very similar results to those from other authors [16]. We believe that the own structural deformity of the fifth metatarsal is the determinant of the medial displacement of the sesamoid in the case where this is present.

## Conclusions

In our population, we have observed a similar percentage of biphalangeal toes, slightly higher than the studies on the caucasic populations. We have showed that fifth triphalangeal toes seems to present with a higher proportion of pathology. The risk of suffering from hammer toe was almost 4 times greater in triphalangeal toes. Among the patients who were operated from the fifth toe there were a higher proportion of triphalangeal toes, but this proportion was lower than in the total population of studied feet.

## Authors’ original submitted files for images

Below are the links to the authors’ original submitted files for images. Authors’ original file for figure 1 Authors’ original file for figure 2 Authors’ original file for figure 3 Authors’ original file for figure 4 Authors’ original file for figure 5 Authors’ original file for figure 6
