# Supplementary figures and images for: Biphalangeal/triphalangeal fifth toe and impact in the pathology of the fifth ray
Source: BMC Musculoskelet Disord. 2014 Sep 5;15:295. doi: 10.1186/1471-2474-15-295 (PMC4161841; doi:10.1186/1471-2474-15-295)

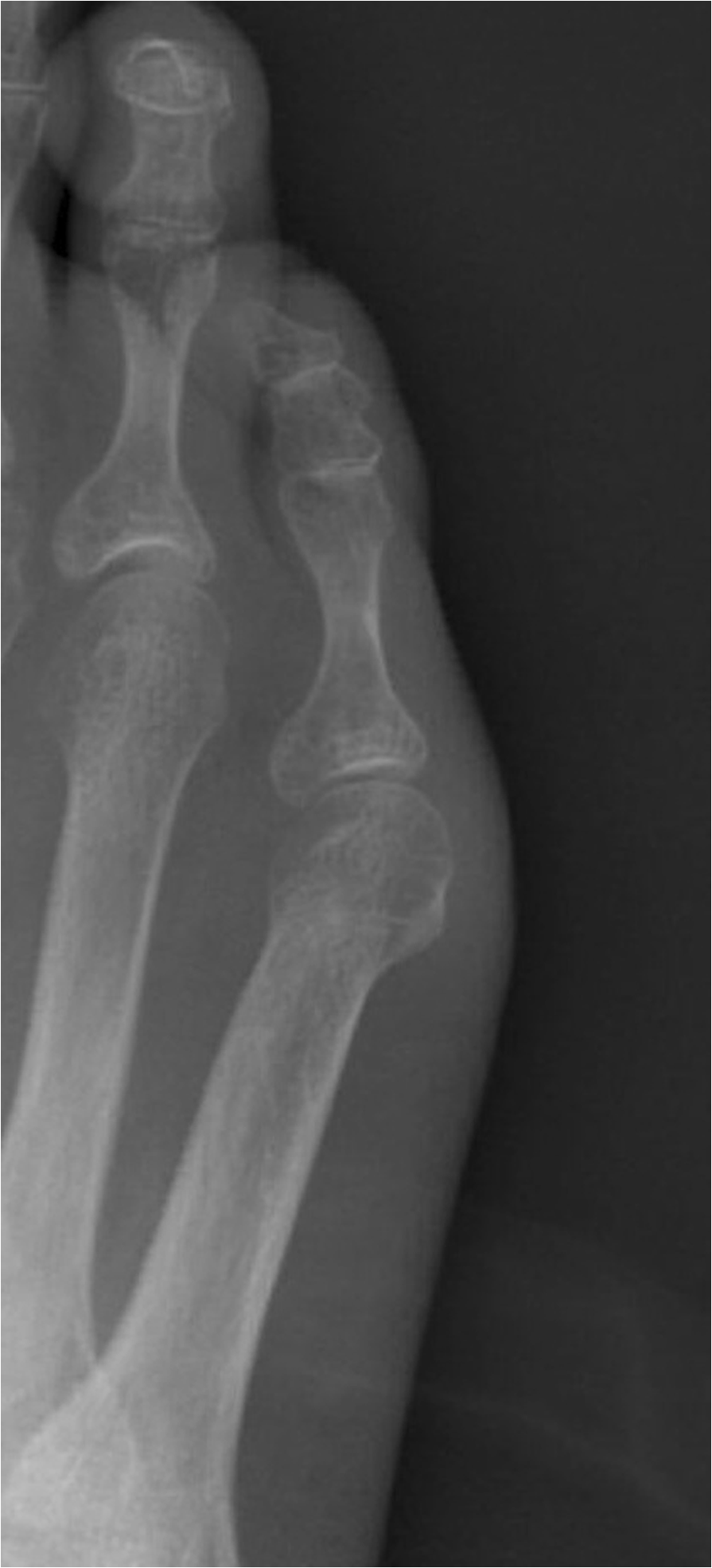

Supplement: Supplementary file 1 — Authors’ original file for figure 1 [file 12891_2013_2235_MOESM1_ESM.tif]

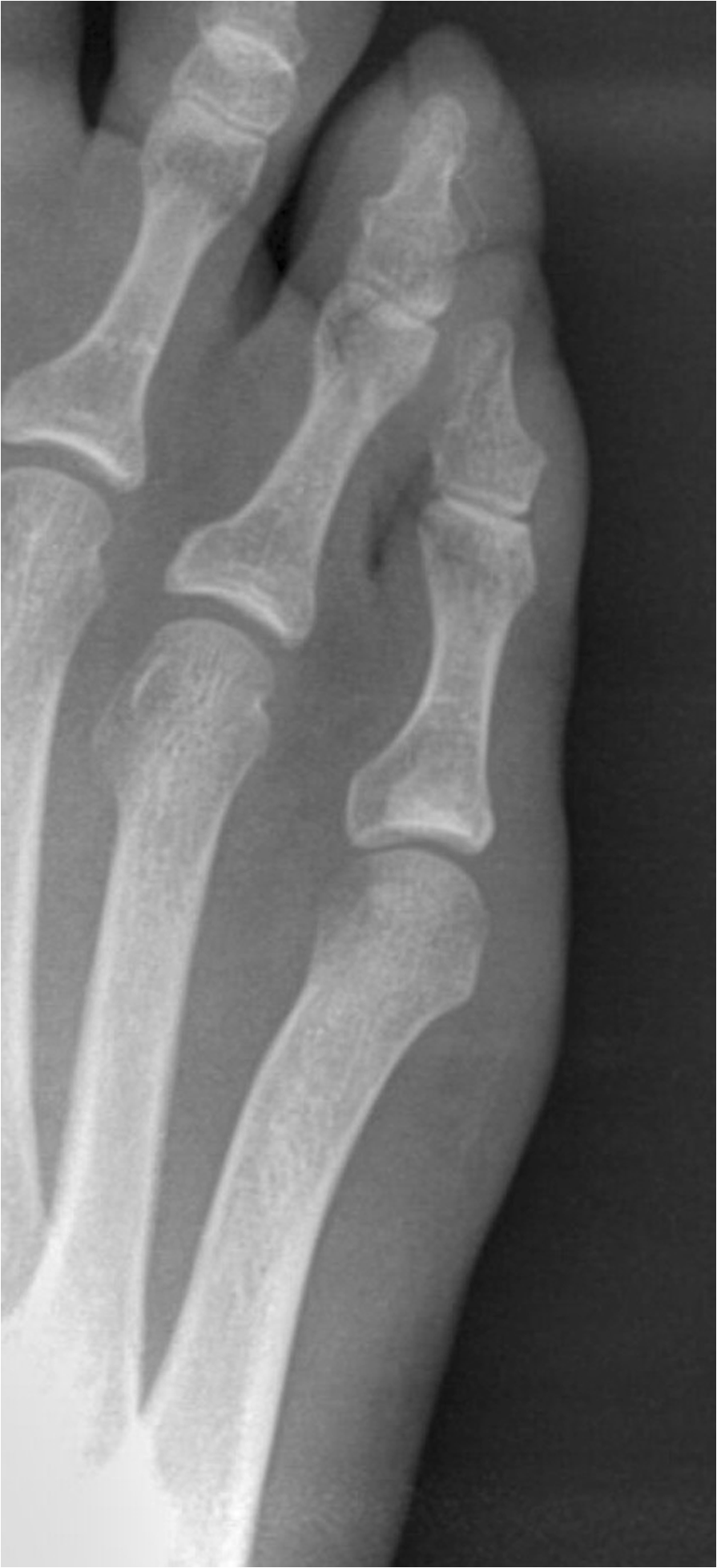

Supplement: Supplementary file 2 — Authors’ original file for figure 2 [file 12891_2013_2235_MOESM2_ESM.tif]

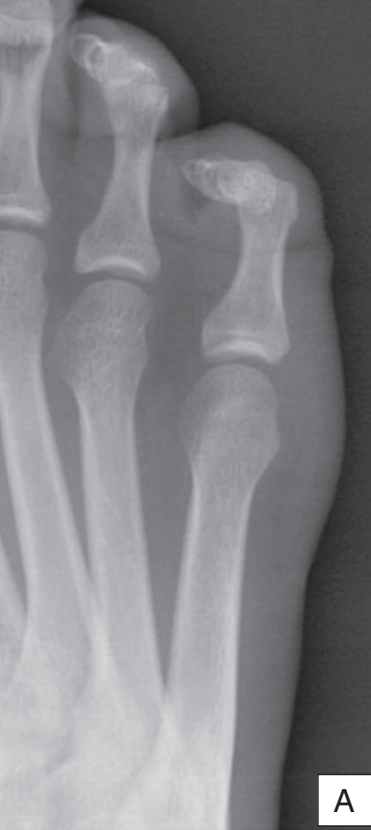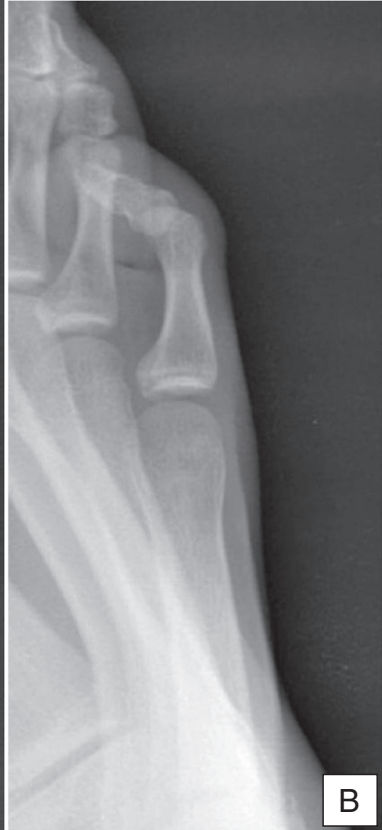

Supplement: Supplementary file 3 — Authors’ original file for figure 3 [file 12891_2013_2235_MOESM3_ESM.pdf]

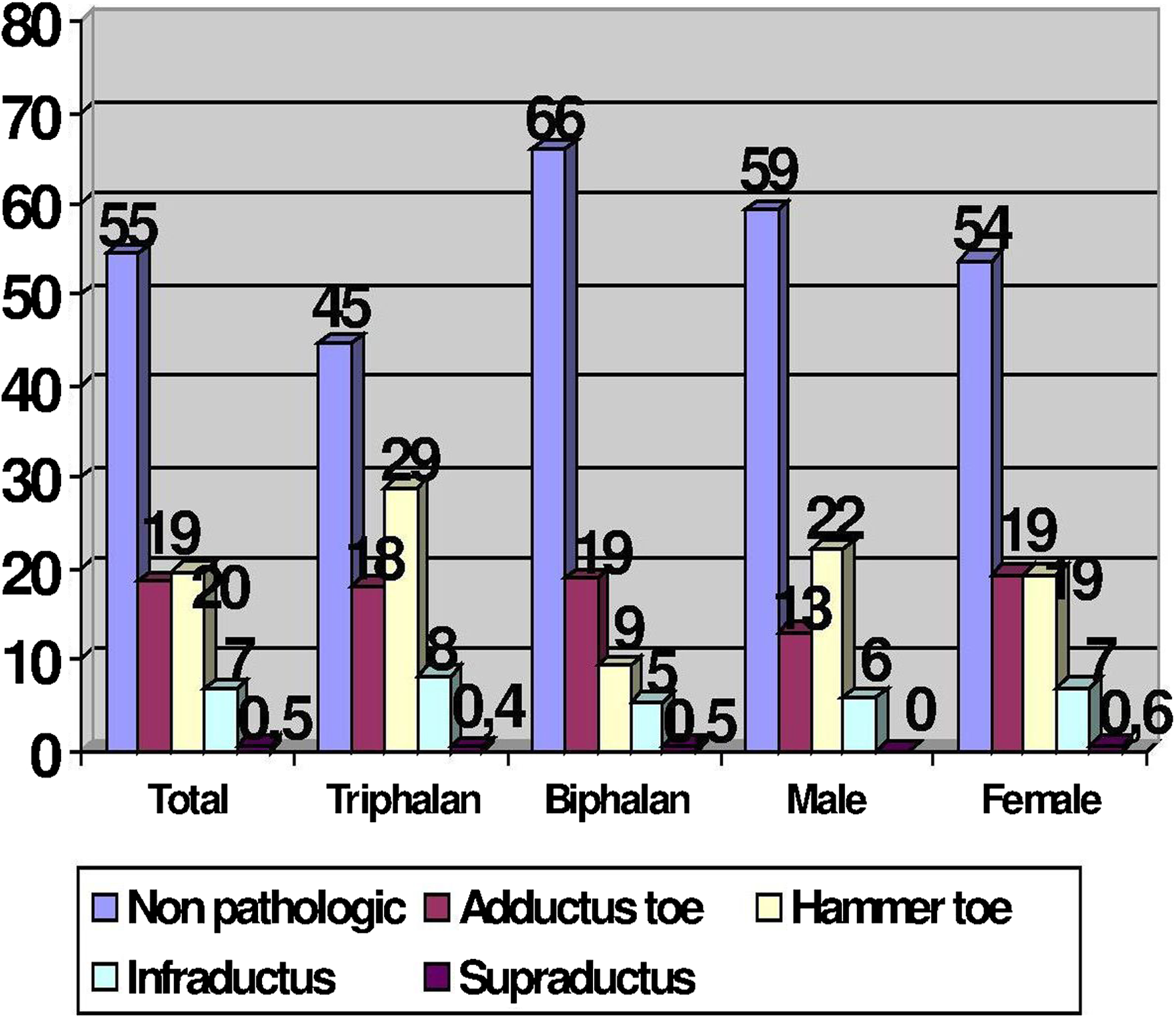

Supplement: Supplementary file 4 — Authors’ original file for figure 4 [file 12891_2013_2235_MOESM4_ESM.tif]

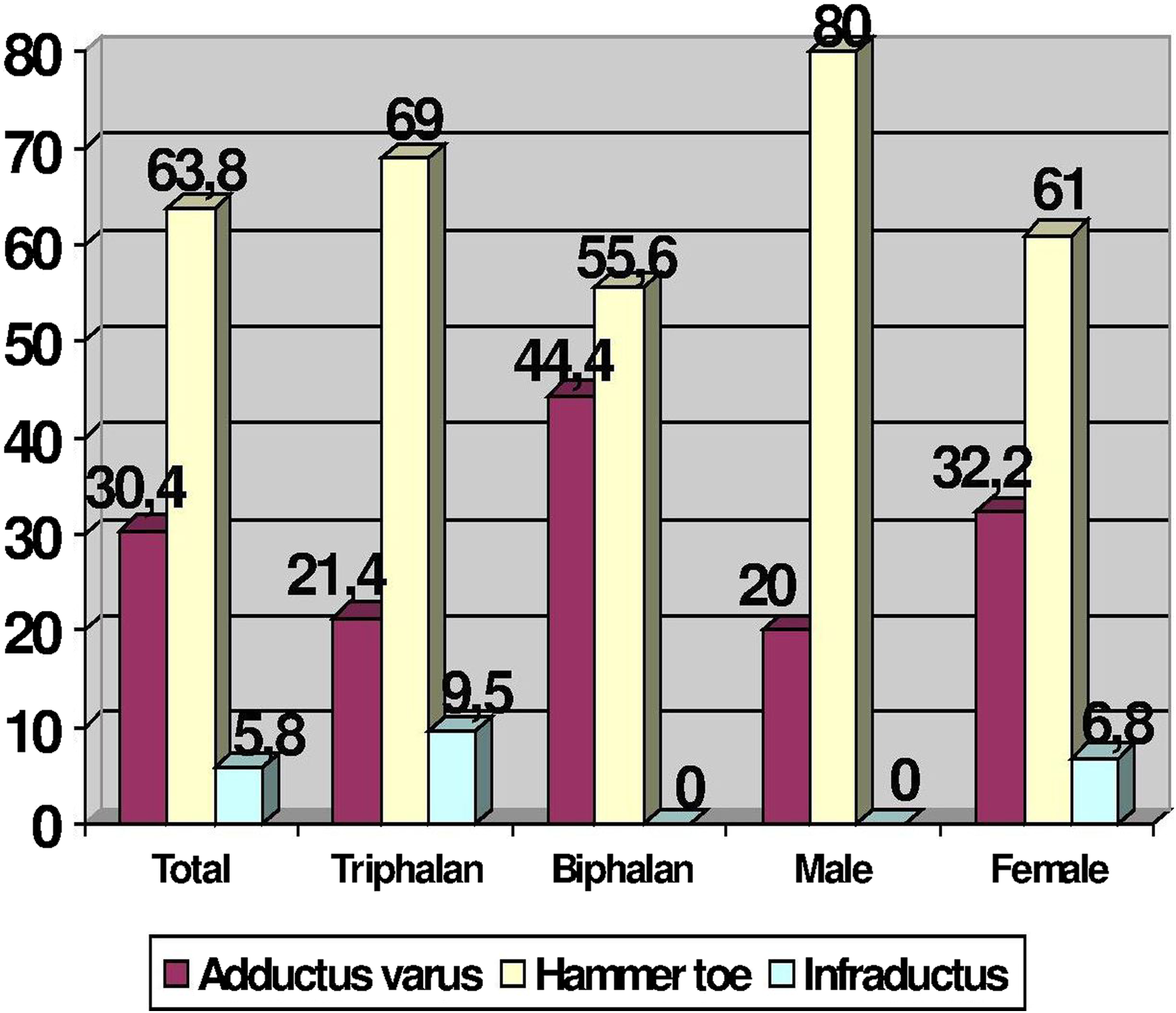

Supplement: Supplementary file 5 — Authors’ original file for figure 5 [file 12891_2013_2235_MOESM5_ESM.tif]

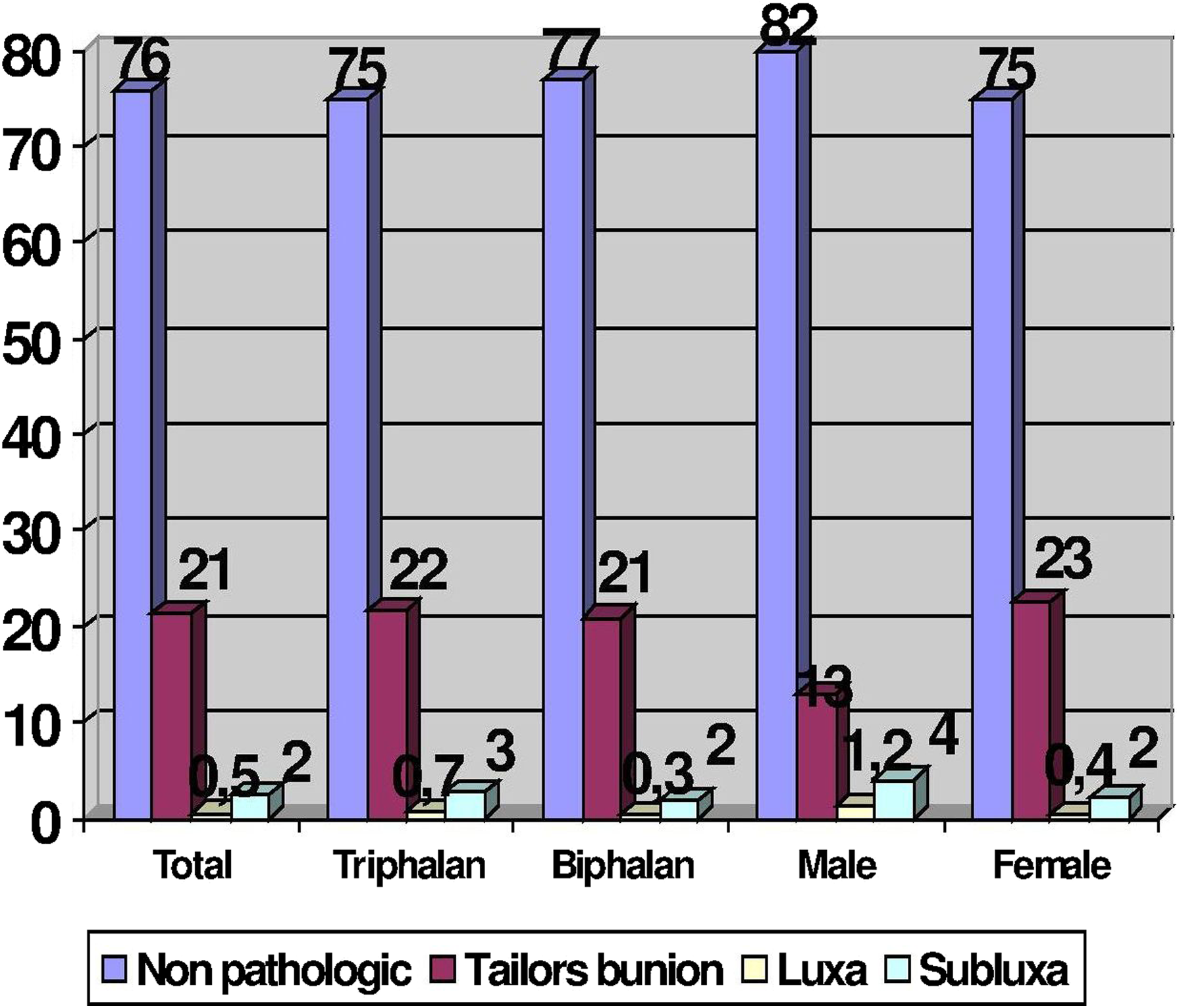

Supplement: Supplementary file 6 — Authors’ original file for figure 6 [file 12891_2013_2235_MOESM6_ESM.tif]
